# Transferrable property relationships between magnetic exchange coupling and molecular conductance†

**DOI:** 10.1039/d0sc04350h

**Published:** 2020-10-08

**Authors:** Martin L. Kirk, Ranjana Dangi, Diana Habel-Rodriguez, Jing Yang, David A. Shultz, Jinyuan Zhang

**Affiliations:** Department of Chemistry and Chemical Biology, The University of New Mexico MSC03 2060, 1 University of New Mexico Albuquerque New Mexico 87131-0001 USA mkirk@unm.edu; Center for High Technology Materials, The University of New Mexico Albuquerque New Mexico 87106 USA; Department of Chemistry, North Carolina State University Raleigh North Carolina 27695-8204 USA shultz@ncsu.edu

## Abstract

Calculated conductance through Au_*n*_–S–Bridge–S–Au_*n*_ (Bridge = organic σ/π-system) constructs are compared to experimentally-determined magnetic exchange coupling parameters in a series of Tp^Cum,Me^ZnSQ–Bridge–NN complexes, where Tp^Cum,Me^ = hydro-tris(3-cumenyl-1-methylpyrazolyl)borate ancillary ligand, Zn = diamagnetic zinc(ii), SQ = semiquinone (*S* = 1/2), and NN = nitronylnitroxide radical (*S* = 1/2). We find that there is a nonlinear functional relationship between the biradical magnetic exchange coupling, *J*_D→A_, and the computed conductance, *g*_mb_. Although different bridge types (monomer *vs.* dimer) do not lie on the same *J*_D→A_*vs. g*_mb_, curve, there is a scale invariance between the monomeric and dimeric bridges which shows that the two data sets are related by a proportionate scaling of *J*_D→A_. For exchange and conductance mediated by a given bridge fragment, we find that the ratio of distance dependent decay constants for conductance (*β*_g_) and magnetic exchange coupling (*β*_J_) does not equal unity, indicating that inherent differences in the tunneling energy gaps, Δ*ε*, and the bridge–bridge electronic coupling, ***H***_BB_, are not directly transferrable properties as they relate to exchange and conductance. The results of these observations are described in valence bond terms, with resonance structure contributions to the ground state bridge wavefunction being different for SQ–Bridge–NN and Au_*n*_–S–Bridge–S–Au_*n*_ systems.

## Abstract 2

Calculated conductance through Au*_n_*–S–Bridge–S–Au*_n_* constructs are compared to experimental magnetic exchange coupling parameters in Tp^Cum,Me^Zn(SQ–Bridge–NN) complexes, where SQ = semiquinone radical and NN = nitronylnitroxide radical.

## Graphical abstract

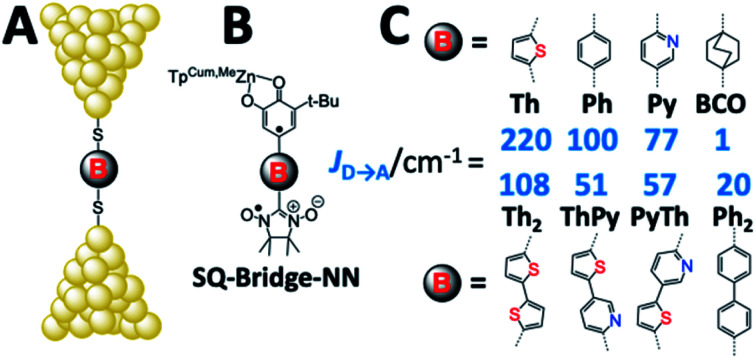

## Introduction

**Fig. 1:**
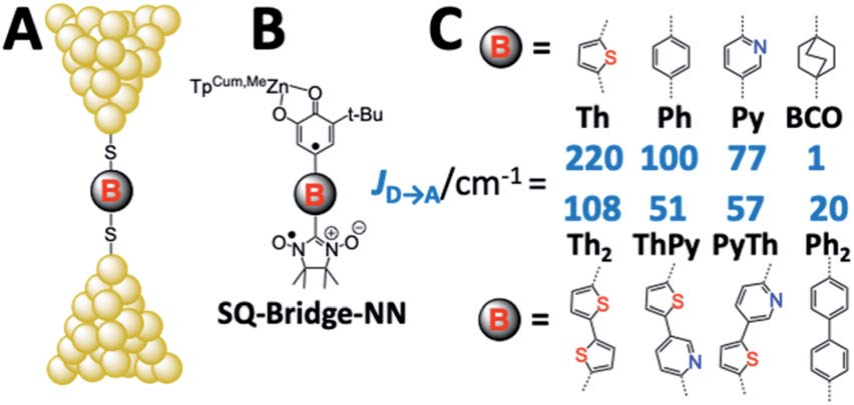
(A) Metal–Bridge–Metal junctions corresponding to biradical complexes (B). (C) Bridges common to M–B–M junctions and Donor–Bridge–Acceptor complexes (B), along with ferromagnetic exchange parameters, *J*_D→A_ for biradical complexes (C). **BCO** = bicyclo[2.2.2]octane, **Ph** = *para*-phenylene, **Th** = 2,5-thiophene, **Py** = pyridine, **Py-Th** = 2-pyridyl-5′-thiophene, **Ph2** = *para*-biphenylene, and **Th2** = 2,5′-bithiophene.

Electron transport in single-molecule devices is typically interrogated *via* a combination of experimental and computational probes of conductance using Metal–Bridge–Metal (M–B–M) junctions (Fig. 1A).^1–14^ To account for inherent variations in molecule–electrode binding geometries, the experimental determination of conductance (*g*_mb_) typically requires hundreds to thousands of individual conductance measurements in order to construct conductance histograms, which allow researchers to determine both conductance distributions and the most probable conductance value.^15^ Since conductance calculations often utilize a single M–B–M geometry and the molecular geometry in the actual junction is unknown, this can make direct comparisons between theory and experiment difficult to interpret. As a result, studies that correlate single-molecule conductance with physical observables such as electron transfer rate constants (*k*_D→A_)^2,4,5,14,16^ and magnetic exchange couplings (*J*_D→A_)^1,17–20^ continue to be a topic of current research. A primary impact of these efforts will be to provide key insight into orbital pathways for charge transport through molecules, which is difficult to achieve using traditional approaches. These correlations, coupled with the determination of transferable property relationships, are expected to facilitate improved molecular design concepts for molecular electronics and spintronics.

The crux of these transferable property relationships was first described by Nitzan,^21^ who derived a relationship between *g*_mb_ and *k*_D→A_ at parity of the molecular bridge. With some approximations, Nitzan was able to show that conductance could be related to the square of the D–A electronic coupling matrix element, ***H***_D→A_^2^ (***H***_D→A_^2^ = |***H***_DB_***H***_BA_|^2^|*G*_B_(*E*)|^2^), for electron transfer (eqn (1)–(4)).^21^1
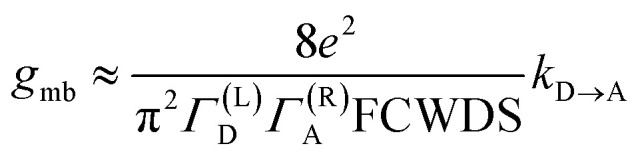
2
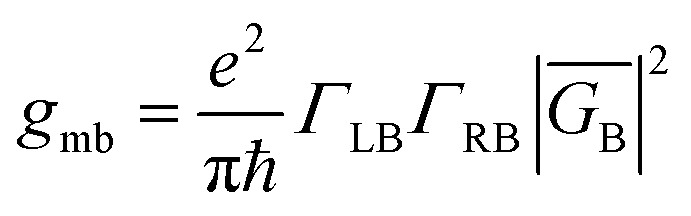
3
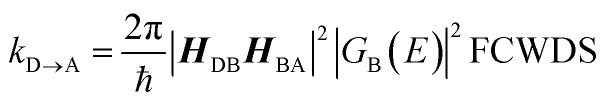
4
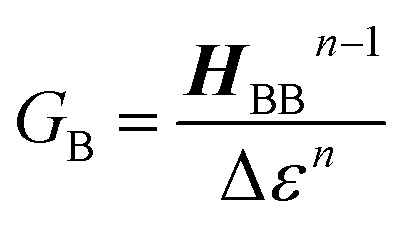
here, the ***H***_*ij*_ are pairwise electronic coupling matrix elements that connect D, B, and A, the *Γ*_*i*_ are broadening functions that represent the coupling between the molecule and electrodes, Δ*ε* is the tunneling energy gap, *G*_B_ is the bridge Green's function with the overbar indicating that *G*_B_ may differ for electron transfer (or magnetic exchange) compared to conductance, and *n* is the number of bridge units. These transferrable property relationships indicate that any physical observable that is a function of ***H***_D→A_^2^ can be related to *g*_mb_ (*e.g.*eqn (5)).

5 *J*_D→A_ ∝ ***H***_D→A_^2^ ∝ *k*_D→A_ ∝ *g*_mb_

This leads to a corresponding relationship between these parameters and the distance decay constants (*β*_i_) for electron transfer, conductance, and magnetic exchange as indicated in eqn (6),

6 *k*_D→A_ ∝ *g*_mb_ ∝ *J*_D→A_ ∝ exp(−*β*_i_ × *nL*)

where *nL* is the distance spanned by the molecular bridge (*e.g. R*_DA_ – *R*_0_).

There is both experimental and computational support for a correlation between *k*_D→A_, *g*_mb_ and *J*_D→A_.^14,17,22–31^ Beratan and Waldeck^4,5^ have built on Nitzan's work to show that the relationship between *k*_D→A_ and *g*_mb_ is nonlinear for alkane and peptide nucleic acid oligomer bridges, but they are related by a power law dependence. Their work supports an argument that the bridge Green's function, *G*_B_, is not the same for electron transfer and conductance. As a result, Beratan and Waldeck^4,5^ were able to show that the distance decay constants for *k*_D→A_ and *g*_mb_ are, in general, inequivalent and a linear relationship between *k*_D→A_ and *g*_mb_ will only occur if the individual *β*_i_ are identical.

Since the magnetic exchange interaction (*J*_D→A_) is proportional to ***H***_D→A_^2^*via*eqn (5),^32–36^ we have initiated experiments designed to explore the correlation of *J*_D→A_ between donor–acceptor biradical centers that are spanned by molecular bridge units (D–B–A biradicals = SQ–Bridge–NN; SQ = *S* = 1/2 semiquinone, and NN = *S* = 1/2 nitronylnitroxide radical, Fig. 1B and C)^1,17–20,26,35–40^ with computed molecular conductance values, *g*_mb_.^1^ An advantage to understanding the magnitude of *g*_mb_ by correlating with experimental *J*_D→A_ parameters is highlighted by the fact that the variable-temperature magnetic susceptibility measurements used to determine *J*_D→A_ are performed on solid-state samples with known geometries that have been determined by X-ray crystallography. Thus, there is a single conformation present that contributes to *J*_D→A_. Unlike experimental conductance measurements in a contact geometry and solution electron transfer rate measurements, the magnitude of *J*_D→A_ determined by solid state magnetic susceptibility measurements is not affected by structural and solvent bath induced inhomogeneities or by decoherence effects.

**Fig. 2:**
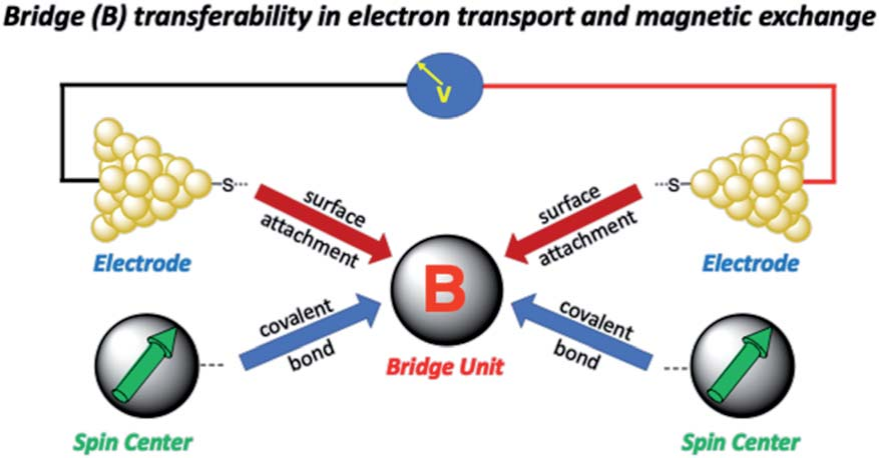
Illustration of how bridge-mediated electronic coupling is related to conductance and magnetic exchange coupling. Top: Molecular bridge (B) connects metallic electrodes to give a Metal–Bridge–Metal device. Bottom: Molecular bridge (B) connects spin centers to facilitate magnetic exchange coupling.

Relating molecular orbital pathways for *J*_D→A_ to orbital transport channels for *g*_mb_ has its own set of challenges, and these include (1) differences in the molecular fragments covalently attached to the bridge unit, (2) the molecular identity of surfactant groups, (3) variations in surface contacts, (4) the choice of electrode material and surface morphology, and (5) inhomogeneities in molecular structure (*e.g.*, conformation) and in contact geometries. As alluded to above, an important related question was recently posited by Herrmann,^14^ which concerns the transferability of geometric and electronic structure contributions of the bridge moiety to *k*_D→A_, *g*_mb_, and *J*_D→A_ (Fig. 2). Herein, we address these difficulties and the concept of bridge electronic structure transferability between conductance and exchange. We then use our results to explicitly determine distance decay constants (*β*_g_) for *g*_mb_ from prior studies that have determined the distance dependence of *J*_D→A_. Our results are described in valence bond terms, with resonance structure contributions to the ground state bridge wavefunction being different for SQ–Bridge–NN and Au_*n*_–S–Bridge–S–Au_*n*_ systems. This provides a path forward for understanding the power law relationship between *g*_mb_ and *J*_D→A_ in the absence of structural inhomogeneities and decoherence effects.

## Experimental

### General

The synthesis and characterization of **SQ–BCO–NN**, **SQ–Ph–NN**, **SQ–Th–NN**, **SQ–Ph2–NN**, **SQ–Py–Th–NN**, and **SQ–Th2–NN** have been published previously.^1,17,20,26,35,39,41,42^ See the ESI† for the preparation and characterization (spectroscopic data, X-ray crystal structure, and magnetic characterization) of the pyridyl-bridged biradical complex, **SQ–Py–NN**.

### Resonance Raman spectroscopy

Solution (methylene chloride) resonance Raman spectra were collected in either a 180° backscattering (780 nm) or 90° scattering geometry using 407 nm excitation from a Coherent Innova 70 Kr^+^ (1 W) ion laser. The scattered radiation was passed through a longpass filter (Semrock RazorEdge) to remove Rayleigh scattered laser light and then dispersed onto a liquid nitrogen-cooled Infrared Associates CCD detector using a Princeton Acton spectrograph. The laser power at the sample was typically kept between 40 and 100 mW in order to prevent possible photo- and thermal degradation of the sample. The sample was sealed in a glass capillary tube and spun with a custom-made sample holder. All data were scan-averaged, and any individual data set with vibrational bands compromised by cosmic events were discarded.

### Molecular electronic structure calculations

Thiophene and benzene calculations were performed at the density functional level of theory using the Gaussian 09W software package.^43^ Input files were prepared using the molecular builder function in GaussView. Calculations employed the B3LYP hybrid exchange–correlation functional and a 6-31g(d′,p′) split valence basis set with polarizability functions was used for all atoms. Frontier molecular orbitals (MOs) were generated from fully optimized ground states.

### Transport calculations

Transport calculations were performed using ATK 2016.0 v16.0,^44–46^ as detailed in our earlier work.^1^ The device configuration consists of the left Au electrode, the molecular bridge (scattering region), and the right Au electrode. Both the left and right gold electrodes consist of nine layers with each layer comprised of a 3 × 3 array of Au(111) atoms, for a total of 81 gold atoms per electrode. For the electrodes, the C or *z* direction is periodic in the system. This is also the direction of electron transport. Initial geometry optimizations of the bridge molecules **BCO**, **Py**, **Ph**, **Th**, **Ph2**, **Py-Th**, **Th2** were performed using Gaussian 09 (ref. 43) with a 6-31g(d′,p′) basis set and the B3LYP functional. Electrode surfaces were constructed by cleaving the bulk crystal and using a copy of this to form the second electrode. The molecule was then placed on the surface of the left electrode and subsequently connected to the right electrode through terminal sulfur atoms to create the final device geometry. Prior to creating the final device geometry, the bulk configuration was optimized using a single-zeta basis set for the gold atoms (to save computational time) and a double zeta basis set for all other atoms. The optimization was performed using ATK-DFT with a Perdew–Zunger local density approximation (LDA-PZ) exchange–correlation. After this bulk optimization, the configuration was converted into a final device geometry for all subsequent calculations. A 5 × 5 × 51 *K*-point sampling has been used in *x*, *y*, *z*-directions, respectively. The boundary conditions were Dirichlet (fixed boundary condition) in the *z* direction, which is the direction of transport. The boundary conditions in the *x* and *y* directions were disabled so they could not be changed but the default parameterization is periodic for the *x* and *y* directions. The NEGF formalism was used to calculate the non-equilibrium electron density of the central region of the device.

The Landauer–Büttiker formula^47^ relates transmission probabilities to conductance, *g*_mb_ (*g*_mb_ = *I*/*V*). This formula is used to calculate the voltage dependent current, *I*(*V*), across a molecular junction, which is determined by integrating the transmission function, *T*(*E*,*V*), according to eqn (7).^47^7
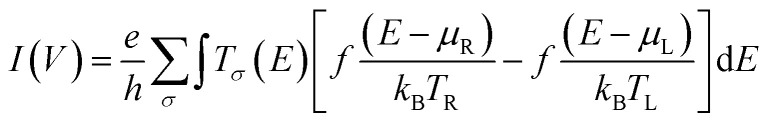
here, the Fermi–Dirac distribution functions for the right and left electrodes are given by *f*(*E* − *μ*_R_)/*k*_B_*T*_R_ and *f*(*E* − *μ*_L_)/*k*_B_*T*_L_, with *μ*_R_ and *μ*_L_ being the chemical potentials for the right and left electrodes, respectively, *e* is the charge of the electron, *h* is Planck's constant, *eV*_bias_ is the bias window equivalent to *μ*_R_–*μ*_L_ (where *V*_bias_ = *V*_L_ − *V*_R_), *T*_*σ*_(*E*) is the transmission coefficient for the spin component, *σ*, describing the junction at an energy *E* and a bias voltage *V*_bias_. All electron transport properties were computed using the ATK software package that includes virtual nanolab associated analysis modules. The molecular projected self-consistent Hamiltonian (MPSH) technique has been used to understand the molecular orbital origin of the resonant peaks in the transmission spectra.^48,49^

## Results and discussion

### Exchange coupling in SQ–Bridge–NN complexes

Magnetic exchange coupling constants (*J*_D→A_) for the monomeric SQ–Bridge–NN biradical complexes (Fig. 1B and C) **SQ–BCO–NN**, **SQ–Ph–NN**, **SQ–Th–NN**,^17,20,26,41^ and for the dimeric bridge molecules **SQ–Py–Th–NN**,^1^**SQ–Ph2–NN**,^17^ and **SQ–Th2–NN**^17^ have previously been determined by magnetic susceptibility measurements. The dimer exchange spin Hamiltonian (eqn (8)):8
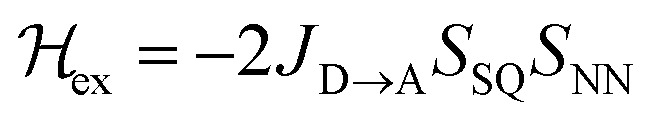
has been used in the analysis of these data, including the analysis of new magnetic susceptibility data for the pyridyl-bridged complex (**SQ–Py–NN**), which yields *J*_D→A_ = +72 cm^−1^ (see ESI†). Collectively, these SQ–Bridge–NN biradical systems possess a large range of *J*_D→A_-values, bridge-dependent electronic structures, and both σ- and π-coupling units. Notably, all of these molecules are *ferromagnetically* exchange coupled^20,35,36^ with *J*_D→A_ values that vary from a low of *J*_D→A_ = +1 cm^−1^ for σ-mediated exchange in **SQ–BCO–NN**, to a markedly larger *J*_D→A_ = +220 cm^−1^ for π-mediated exchange in **SQ–Th–NN**.

### Exchange coupling within the context of a valence bond configuration interaction model

**Fig. 3:**
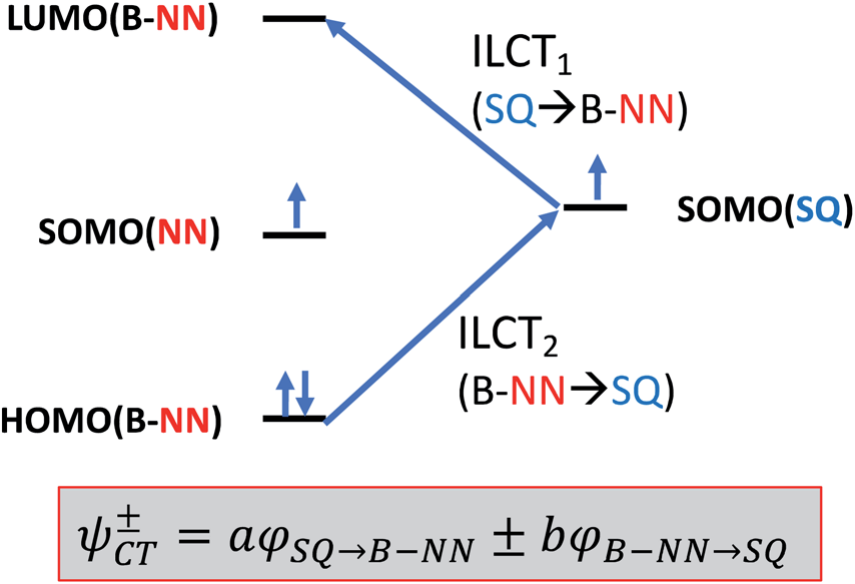
Simple 4-orbital diagram showing NN–Bridge(HOMO) → SQ(SOMO) and SQ(SOMO) → NN–Bridge(LUMO) one-electron promotions that figure prominently in charge transfer configurations that contribute to magnetic exchange in SQ–Bridge–NN biradicals.

**Fig. 4:**
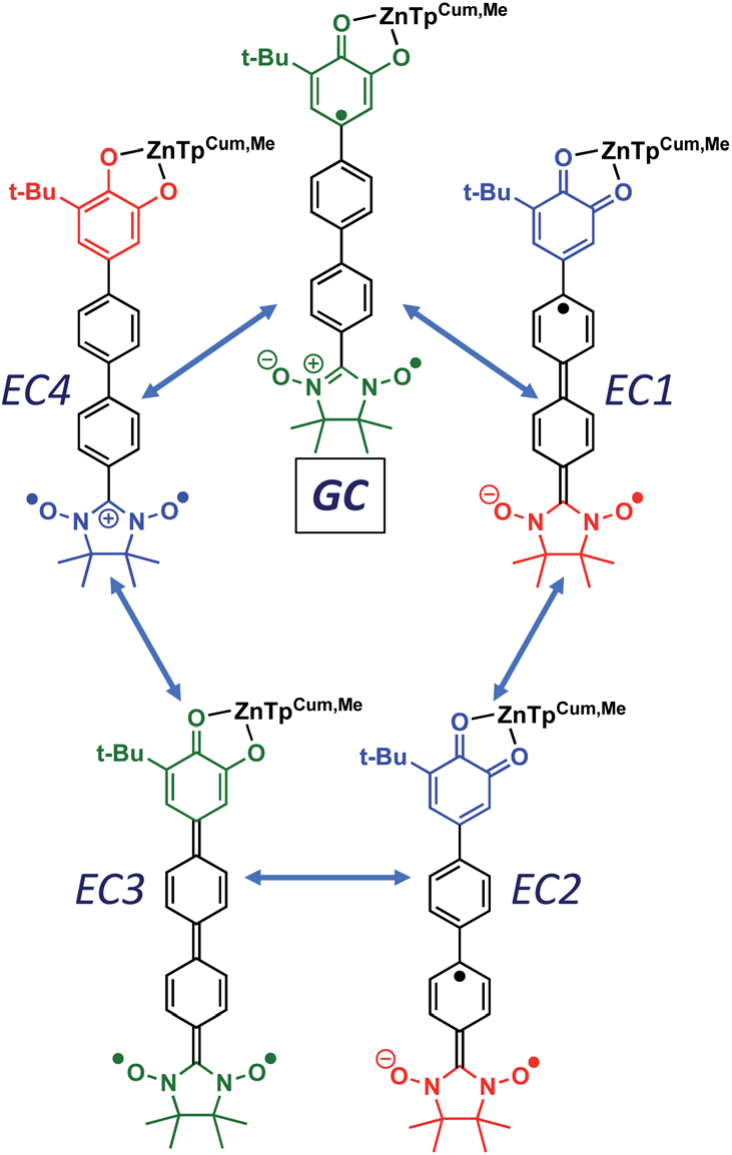
Resonance structures illustrating enhanced ***H***_BB_, ***H***_DB_ and ***H***_BA_ couplings in D–B–A biradicals. The corresponding zwitterionic and biradical resonance structures for M–B–M couplings (***H***_BB_, *Γ*_LB_, *Γ*_RB_) are higher in energy and contribute less to the conductance. All SQ–B–NN ligands have a net −1 charge. Donor and acceptor moieties are color-coded according to oxidation state: green = 9 π-electron SQ^1−^/7 π-electron NN; red = 10 π-electron catecholate^2−^/8 π-electron NN^1−^; blue = 8 π-electron quinone^0^/6 π-electron NN^1+^.

Given the geometric structures of these SQ–Bridge–NN biradicals, we can conveniently divide them into two distinct bridge categories: the first possesses single-ring molecular bridges, while the second contains two-ring molecular bridges where each bridge ring is connected to the other *via* a σ-bond. The electronic origins of π-mediated exchange coupling in **SQ–Ph–NN** and σ-mediated exchange in **SQ–BCO–NN** have been rigorously evaluated using a combination of spectroscopy, magnetic susceptibility, and theory.^19,20,26,39,41,50^ For π-mediated exchange, a valence-bond configuration interaction (VBCI) model has been used.^19,20,35,36^ Within the context of more traditional orbital pathways for understanding exchange coupling, the VBCI method conveniently illustrates how specific excited state configurations admix with the ground configuration to affect the magnitude of *J*_D→A_. The NN(SOMO), SQ(SOMO), NN–Bridge(HOMO), and the NN–Bridge(LUMO) (Fig. 3) orbitals form a convenient minimal active space to generate the ground and excited state configurations that contribute to *J*_D→A_ for *conjugated* SQ–Bridge–NN systems. Previous work has demonstrated the crucial role of the LUMO(B–NN) in determining the magnitude of *J*_D→A_.^17,19,20,35,36,39^ Resonance structures that illustrate both the spin and the charge distributions of ground state **SQ–Ph2–NN** configurations and excited state configurations that arise from one-electron promotions within this active space are shown in Fig. 4. Here, the ground SQ–Bridge–NN configuration is represented by GC, EC1 and EC2 represent the configuration that results from an intraligand SQ(SOMO) → NN–Bridge(LUMO) electron promotion, and the NN–Bridge(HOMO) → SQ(SOMO) one-electron promotion is represented by the EC3 and EC4 resonance structures. Similar resonance structures can be drawn for other SQ–Bridge–NN biradical complexes. For the *conjugated* SQ–Bridge–NN systems discussed here, the charge transfer configuration generated by the intraligand SQ(SOMO) → NN–Bridge(LUMO) one-electron promotion (EC1 and EC2) has been shown to be the dominant contributor to the ground state *ferromagnetic* exchange coupling in **SQ–Ph–NN**, with the NN–Bridge(HOMO) → SQ(SOMO) (EC3 and EC4) playing a less important role.^17,19,20,35,36,39^ The relative contributions of these two intraligand charge transfer (ILCT) configurations will be dependent on the nature of the bridge. The use of more electron donating bridge molecules (*e.g.*, thiophene) is expected to increase the relative NN–Bridge(HOMO) → SQ(SOMO) charge transfer contribution to the magnetic exchange by raising the energy of the bridge HOMO and LUMO orbitals. In addition, we have shown that bond torsions which disrupt Ph–NN π-coupling result in the ILCT transition being shifted to higher energy, with a concomitant reduction in the ILCT intensity relative to configurations that possess more planar Ph–NN conformations.^41^ As a result, an ILCT transition is *not* observed in **SQ–BCO–NN** and the lack of a CT feature in the visible region of the electronic absorption spectrum correlates with the weak σ-mediated exchange coupling.^26,41^ Our understanding of how the bridge HOMO and LUMO orbitals affect the magnitude of both *J*_D→A_ and *H*_D→A_ allow us to begin an assessment of how these orbitals contribute to the transferability of *J*_D→A_, *k*_D→A_, and *H*_D→A_ to conductance in a biased electron transport configuration (Fig. 2).

### Correlation of exchange and conductance

**Fig. 5:**
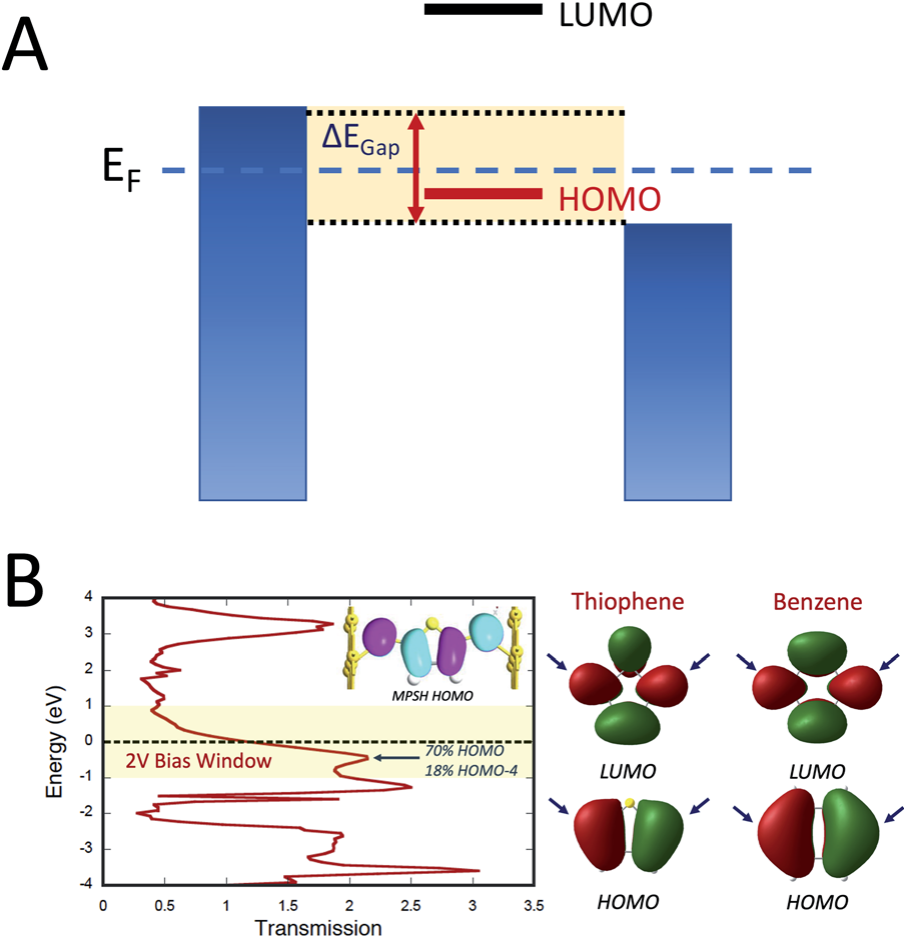
(A) Illustration of the HOMO conductance mechanism. The biased electrodes (blue) are filled to the chemical potential of the electrodes and the variable bias window (Δ*E*_Gap_ = *V*_bias_) is highlighted in yellow. Only the HOMO is dominant in the bias window and therefore it maximally contributes to *g*_mb_. The LUMO is at higher energy and lies outside of the bias window. As such, it does not contribute to *g*_mb_. (B) Left: Computed zero-bias energy *vs.* transmission plot for S–**Th**–S connected to Au electrodes. A 2 V bias window is depicted in yellow and the Fermi energy is shown as a dashed line. Inset: Molecular projected self-consistent Hamiltonian (MPSH) state that dominantly contributes to *g*_mb_, which is primarily comprised of the **Th** HOMO. Right: HOMO and LUMO of **Th** and **Ph**. Bridge carbon atoms of contact are indicated by blue arrows.

Electron transport calculations have been performed on M–B–M constructs (Au_*n*_–S–Bridge–S–Au_*n*_) in order to compare the magnitude of bridge-mediated *J*_D→A_ with *g*_mb_ (*i.e.*, current). In the transport computations, the bridge molecules in Fig. 1C are connected to voltage-biased Au electrodes *via* thiol anchoring groups. Thus, the bridges are the molecular fragments depicted in Fig. 1C with sulfur atoms attached to the bridge carbons. These calculations treat the M–B–M ensemble at the SCF level with the computational results being interpreted in terms of the Landauer–Büttiker formula, which relates transmission probabilities to conductance, *g*_mb_, according to eqn (7).^47^ In marked contrast to the dominant role of the bridge LUMO in SQ–Bridge–NN biradical magnetic superexchange coupling, the dominant orbital contributions to our computed conductance values are the bridge HOMOs. This is clearly illustrated in Fig. 5, where the bias-dependent transmission coefficient for M–Th–M (note that Th is a dithiol in the M–B–M construct) is largest for occupied energy levels that are comprised of thiophene bridge HOMOs. This derives from the bridge HOMOs being effectively pinned to the electrode Fermi level.^48,51,52^ The molecular HOMO character is clearly present in the molecular projected self-consistent Hamiltonian (MPSH) state contributions to the transmission at energies just below the Fermi level for M–Th–M and M–Ph–M (Fig. 5 and S3†). Closer inspection of the MPSH HOMO reveals the presence of anchor sulfur p-orbital contributions, which represent a key difference in the nature of the orbitals that promote M–B–M conductance *vs.* SQ–Bridge–NN magnetic exchange.

Following the work of Beratan,^53^ Yoshizawa and coworkers^54,55^ have conveniently related the zeroth order Green's function for the bridge 
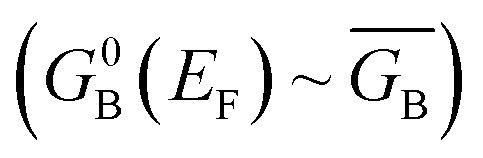
 to the MO coefficients of the bridge orbitals, the Fermi energy, and the individual MO energy levels according to:9
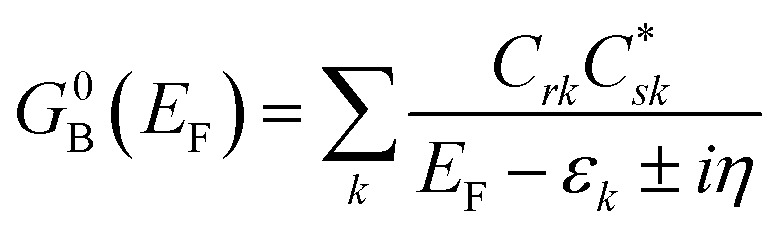
here, the *C*_*rk*_ and 
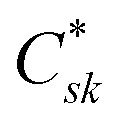
 correspond to the MO coefficients for the sulfur atoms located on the left- and right-hand side of the bridge molecule that directly connects to the electrodes. We note that these sulfur atoms are connected to the same carbon atoms of the organic bridge as the SQ-donor and NN-acceptor moieties in our SQ–Bridge–NN biradical systems (Fig. 5B, right). The sulfur orbital contributions to the MPSH states are important in modulating the conductance, since they directly connect the electrodes to the molecule and allow for delocalization of the bridge wavefunction in the scattering region.^55^ When considering only the HOMO contributions to the conductance, eqn (9) reduces to:^55^10
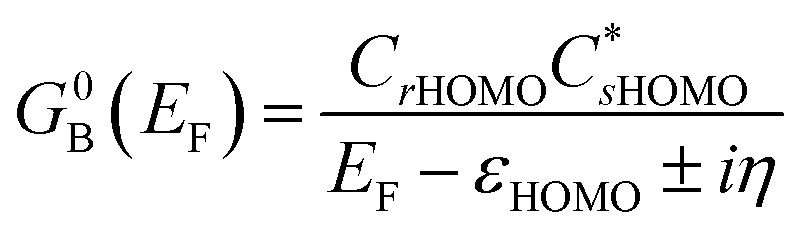


Thus, our *a priori* expectation is a difference in bridge-mediated coupling for conductance and magnetic exchange that results from the importance of sulfur atom character in the M–B–M MPSH HOMOs and the importance of the bridge LUMO that facilitates magnetic exchange in SQ–Bridge–NN biradicals.

**Fig. 6:**
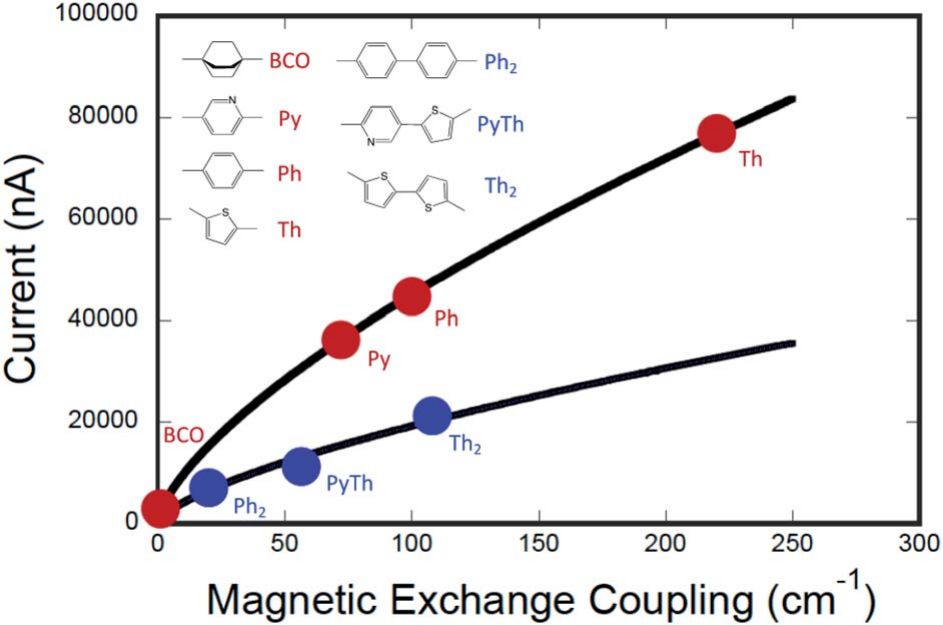
Computed current (*g*_mb_ = *I*/*V*) *vs.* exchange coupling (*J*_D→A_) for monomeric molecular bridges (red circles) and for dimeric molecular bridges (blue circles). The data indicate that current is not directly proportional to magnetic exchange coupling. The data are fit to separate empirical power law functions with identical exponents, with best fits shown as black lines. Computations were performed using a bias voltage of +2 V which approximates the intraligand charge transfer energy in SQ–Bridge–NN complexes.

The computed conductance as a function of the experimentally determined SQ–Bridge–NN magnetic exchange coupling constants using monomeric and dimeric bridge molecules is presented in Fig. 6. We have fit these data to the power law expression:

11 *g*_mb_ = *a*(*cJ*^0^_D→A_)^*γ*^

where *J*^0^_D→A_ is the exchange parameter for SQ–NN with no bridge (*J*^0^_D→A_ = +550 cm^−1^).^19,35,36^ The best fit to the data shown in Fig. 6 yields *a* = 2012 and *γ* = 0.675. One immediately notices two important aspects of this *J*_D→A_*vs. g*_mb_ correlation. The first is that although there is a functional relationship between *J*_D→A_ and *g*_mb_, it is not a linear relationship (*i.e.*, *γ* ≠ 1). The second observation is that the data for different bridge types (monomer *vs.* dimer) do not lie on the same curve. However, the fits to the data do indicate that there is a scale invariance that relates *J*_D→A_ and *g*_mb_, for monomeric and dimeric bridges (*c* = 1 and *c* = 0.28 for monomeric and dimeric bridges, respectively), showing that the two data sets are related by a proportionate scaling of the exchange interaction, *J*_D→A_. As a result, we find that *J*_D→A_ and *g*_mb_ are indeed highly correlated amongst this bridge set, but the simple proportionality relationship, *J*_D→A_ ∝ ***H***_D→A_^2^ ∝ *g*_mb_, is not generally valid across a series of monomeric or dimeric bridge molecules.

**Fig. 7:**
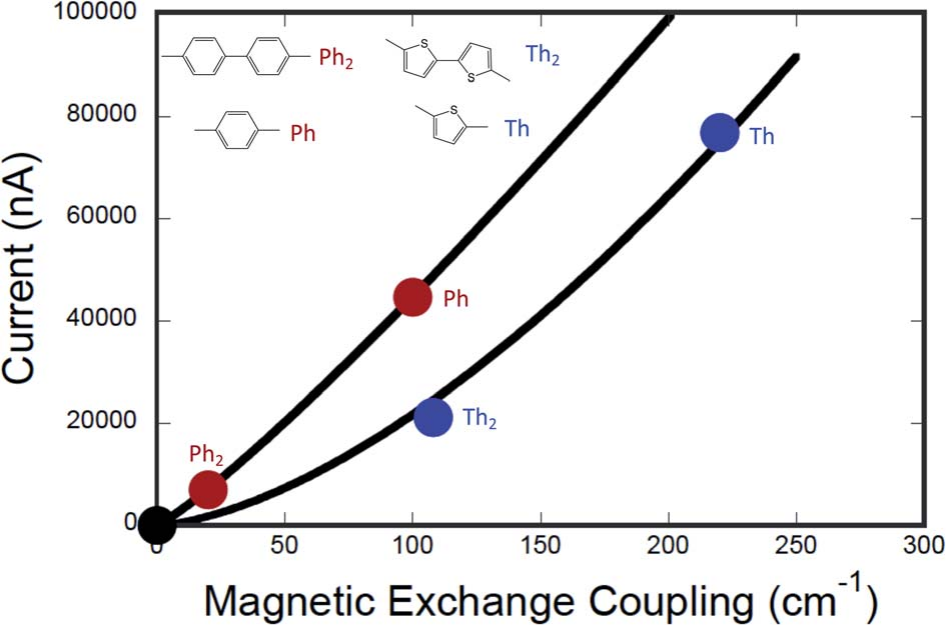
Computed current (conductance) *vs.* exchange coupling for phenylene bridges (red circles) and for thiophene bridges (blue circles). Best fits to the Eq. in the text are shown as red and blue lines, respectively. Note that the value of the exponents, *m* = *β*_g_/*β*_J_, lead to a greater degree of linearity for the phenylene data set (see text). The black circle is located at (0,0), since *g*_mb_ → 0 as *J*_D→A_ → 0. Conductance values for M–B–M systems were computed at +2 V bias using the Landauer–Büttiker formalism, which relates transmission probabilities to conductance (*g*_mb_ = *I*/*V*).

Fig. 7 shows the computed current as a function of magnetic exchange coupling for thiophene and phenylene bridges (0–2 bridge units). Following the formalism of Beratan and Waldeck,^5^ these data have been fit to eqn (12),

12 *g*_mb_ = [*g*_L=0_(*J*^0^_D→A_)^−*β*_g_/*β*_J_^](*J*_D→A_)^*β*_g_/*β*_J_^

in order to understand the relative ratios of distance dependent decay constants for conductance (*β*_g_) and magnetic exchange coupling (*β*_J_). The best fits of eqn (12) to our *g*_mb_*vs. J*_D→A_ data yield *β*_g_/*β*_J_ = 1.15 for the phenylene series and *β*_g_/*β*_J_ = 1.58 for the thiophene series (Fig. 7). Beratan and Waldeck also observed a nonlinear relationship between *g*_mb_ and heterogeneous electron transfer rate constants, *k*_ET_.^4,5^ However, in their study they showed that *β*_g_/*β*_ET_ < 1 for a series of alkanethiol and peptide nucleic acid oligomer bridges, with electron transfer decaying more rapidly with increasing bridge distance than conductance. Here, we observe the opposite correlation, with conductance falling off more rapidly with distance than the magnetic exchange coupling (*β*_g_/*β*_J_ > 1), albeit using very different molecular bridges.

The relationship between *g*_mb_ and *J*_D→A_ for thiophenes is markedly more nonlinear than what we observe in the phenylene data. Eqn (13) describes how differences in these exponential13
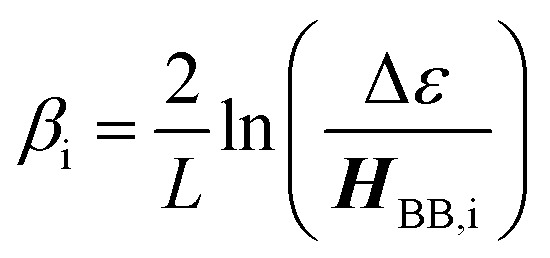
decay constants (*β*_i_) directly relate to inherent differences in the tunneling energy gaps, Δ*ε*, the bridge–bridge electronic coupling, ***H***_BB_, and *L*, the length of the bridge unit.^56,57^ The tunneling energy gap (Δ*ε*) is defined as the energy required to promote an electron from the donor (anode) to the bridge LUMO, or to promote a hole from the acceptor (cathode) to the bridge HOMO, and the distance between the donor and acceptor (or the electrodes) is given by *R*_DA_ (*R*_DA_ = *R*_0_ + *nL*). Thus, a combination of large ***H***_BB_ and a small Δ*ε* lead to low *β*-values and more shallow distance decays that promote long range magnetic superexchange coupling and conductance. Eqn (14) relates these distance decay constants to effective barrier heights, Δ*E*_eff_, with *m*_e_ being the mass of the tunneling electron.^56,57^14
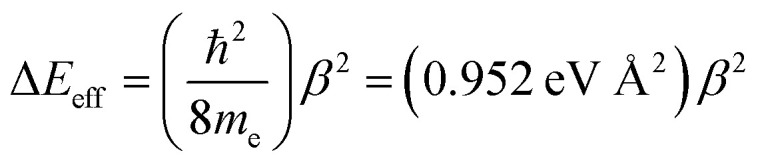


Beratan and Waldeck elegantly attributed their observed deviations in *β*_g_/*β*_ET_ from unity to a combination of charge transfer energy barrier differences between electron transfer in D–B–A ensembles and electron transport in M–B–M configurations, bath-induced decoherences on the bridge molecule, and bridge–bridge electronic coupling (***H***_BB_).^4,5^ Their work comparing conductance values and electron transfer rate constants indicated that differences in effective barrier heights derive from differences between the electrode work function in a transport geometry and donor/acceptor redox potentials in D–B–A systems.^4,5^ With respect to *β*_i_ values, differences in the work function and redox potentials derive from different values for Δ*ε*. In general, due to the interdependence of Δ*ε* and ***H***_BB_ on *β*_i_ values it is difficult to separate these individual contributions to Δ*E*_eff_.^58^

Since decoherence effects are not important for the *J*_D→A_*vs. g*_mb_ correlation presented here, the dominant contributors to the nonlinearity between *J*_D→A_ and *g*_mb_ must relate to differences in bridge–bridge electronic coupling (***H***_BB_) and the tunneling energy gaps (Δ*ε*). For our SQ–Bridge–NN biradical complexes, we have used the ILCT energy as an approximation to the tunneling energy gap. This ILCT transition energy *increases* going from **SQ–Ph–NN** to **SQ–Ph2–NN** and *decreases* when going from **SQ–Th–NN** to **SQ–Th2–NN**.^17^ This contributes to the observed differences in *β*_J_ between **SQ–(Ph)n–NN** and **SQ–(Th)n–NN**, but also contributes to the magnitude of *β*_g_/*β*_J_ and differences in Δ*E*_eff_ for magnetic exchange coupling and conductance.

Previously, we used magnetic susceptibility measurements to determine Δ*E*_eff_ for superexchange-mediated magnetic coupling in SQ–Bridge–NN biradical systems, and this yielded Δ*E*_eff_ = 1173 cm^−1^ and 373 cm^−1^ for phenylene and thiophene bridges, respectively.^17^ Given the conductance to magnetic exchange distance decay ratios (*β*_g_/*β*_J_) for phenylenes and thiophenes determined here, we compute conductance barrier heights (Δ*E*_eff_) of 1555 cm^−1^ and 941 cm^−1^ for phenylene and thiophene bridges that span electrodes in a transport geometry. This translates to a decrease in Δ*E*_eff_ for magnetic exchange coupling of ∼25% (phenylenes) and ∼60% (thiophenes) when compared to conductance values using these same organic bridge fragments. Our *β*_J_ values determined from magnetic susceptibility experiments (*β*_J_ = 0.39 Å^−1^ (Ph_*n*_); *β*_J_ = 0.22 Å^−1^ (Th_*n*_))^17^ and the magnitude of *β*_g_/*β*_J_ determined in this work reveal the corresponding *β*_g_ values for conductance using these molecular bridges (Table 1). The *β*_g_ = 0.45 Å^−1^ value we determine for *para*-phenylene bridges is very close to the *β*_g_ = 0.42 Å^−1^ value determined by conducting probe atomic force microscopy on oligo(*para*-phenylene)-monothiols suspended between metal contacts.^59^ Additionally, the *β*_g_ = 0.35 Å^−1^ value that we have determined for oligothiophene bridges is also in good agreement with conductance studies performed on thiophene bridges with repeat units (*n*) equal to 1, 2, 3 and 5 (*β*_g_ = 0.29 Å^−1^).^60^

**Table 1:** Comparison of decay constants, barrier heights, tunneling energy gaps, and bridge–bridge coupling ratios^a^

Compound/device	Parameter	Value
M–Ph_*n*_–M^b^	*β*_g_	0.45 Å^−1^
	Δ*E*_eff_	1555 cm^−1^
	Δ*ε*_g_/***H***_BB,g_	2.63 (>Δ*ε*_J_/***H***_BB,J_)^c^
**SQ–Phn–NN** b ^,^^17^	*β*_J_	0.39 Å^−1^
	Δ*E*_eff_	1168 cm^−1^
	Δ*ε*_J_/***H***_BB,J_	2.31
M–Th_*n*_–M^b^	*β*_g_	0.35 Å^−1^
	Δ*E*_eff_	941 cm^−1^
	Δ*ε*_g_/***H***_BB,g_	1.79 (>Δ*ε*_J_/***H***_BB,J_)^c^
**SQ–Thn–NN** b ^,^^17^	*β*_J_	0.22 Å^−1^
	Δ*E*_eff_	372 cm^−1^
	Δ*ε*_J_/***H***_BB,J_	1.53

a Data calculated using distances *R*_0_ from X-ray crystal structures of SQ–Bridge_*n*_–NN with one single bond included = 4.30 Å (Ph) and 3.87 Å (Th).

b *n* = 1 and 2.

c Consistent with ***H***_BB,J_ > ***H***_BB,g_ and/or Δ*ε*_g_ > Δ*ε*_J_.

### Resonance contribution to bridge mediated conductance and exchange

Since *β*_i_ and Δ*E*_eff_ are also a function of ***H***_BB_, the bridge–bridge electronic coupling, differences in the nature of the frontier bridge MOs that promote both *g*_mb_ and *J*_D→A_ will also affect the magnitude of the distance decay constants. It is generally assumed that the bridge–bridge electronic couplings encountered in electron transfer, conductance, and magnetic exchange are equivalent, and the donor, acceptor, and electrode contacts do not affect the magnitude of ***H***_BB_. However, this assumption may not be true. A convenient vehicle for understanding electronic structure contributions to *β*_i_ values is the use of contributing resonance structures to highlight excited state configurations that mix with the ground state configuration within a valence bond framework.^41,61–64^

**Fig. 8:**
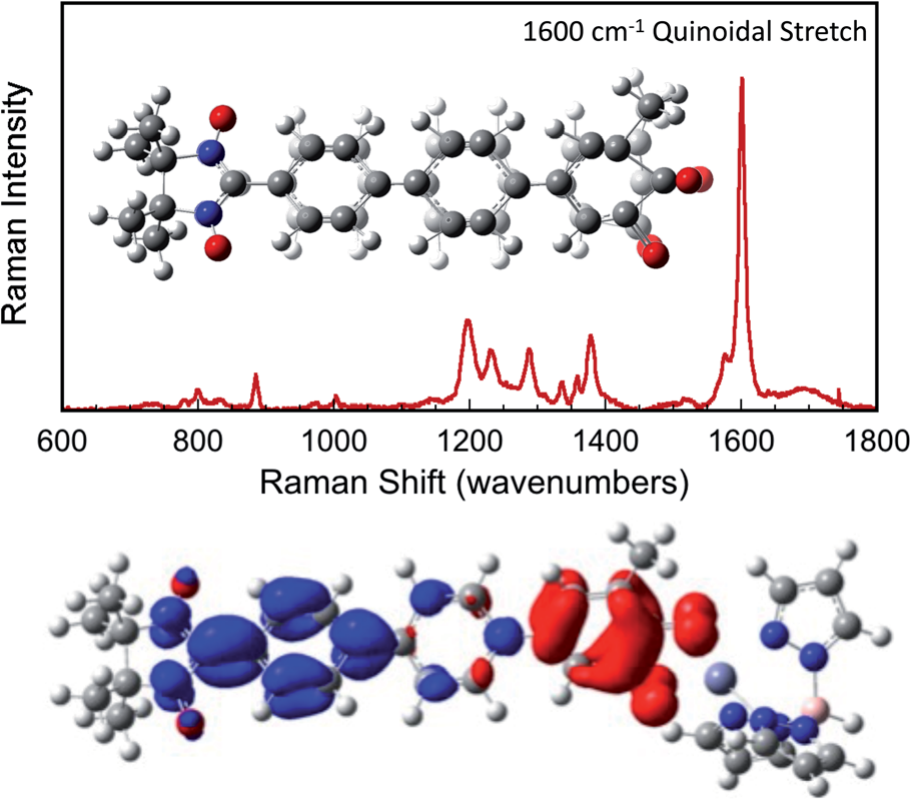
Top: Resonance Raman spectrum collected on resonance with the SQ → Ph–NN ILCT band showing large resonance enhancement of the 1600 cm^−1^ quinoidal stretch (inset). Bottom: Computed electron density difference map (EDDM) that highlights the nature of the SQ → Ph–NN ILCT. Electron density loss in the transition is shown in red, while the electron density gain is shown in blue.

As introduced above, the resonance structures shown in Fig. 4 describe the charge and spin distributions of the ground state configuration (GC) and four excited state configurations (ECs) for the **SQ–Ph2–NN** biradical complex. Quinoidal EC1 and EC3 possess enhanced Ph–Ph π-coupling, which provides a mechanism for increasing the magnitude of ***H***_BB_. Contributions of resonance structures EC1–EC4 to the ground state all lead to enhanced magnetic exchange coupling in SQ–Bridge–NN biradicals. This occurs *via* a combination of B–B, D–B, and B–A electronic couplings (***H***_BB_, ***H***_DB_, ***H***_BA_), which are different than those provided by the coupling of these same bridge molecules with the electrodes (
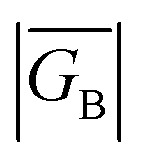
, *Γ*_LB_, *Γ*_RB_). This is partly due to the fact that only high energy zwitterionic and biradical resonance structures can be drawn for the S–Ph–Ph–S electrode linkage. In contrast, these CT configurations lead to a low-energy ILCT state at ∼24 000 cm^−1^ in SQ–Bridge–NN biradicals, which has been shown to admix with the electronic ground state by resonance Raman spectroscopy.^20,41^ Specifically, the effect of this enhanced electronic coupling has been observed in SQ–Bridge–NN biradical compounds by optical pumping into the excited state ILCT and observing a large resonance enhancement of the phenylene quinoidal stretching vibration, which decreases in intensity with increased SQ–Bridge and Bridge–NN bond torsions.^41^ For di-bridged **SQ–Ph2–NN**, a large resonance enhancement of the 1600 cm^−1^ quinoidal stretch is also observed when pumping into the SQ → Ph–NN ILCT band^17^ (Fig. 8). The nature of the charge transfer is indicated by the computed electron density difference map (EDDM) in Fig. 8, which shows net charge transfer from the SQ donor (red) to the Ph–NN acceptor fragment (blue). Thus, the nature of the charge transfer in di-bridged **SQ–Ph2–NN** is analogous to what has been observed previously in the mono-bridged **SQ–Ph–NN** biradical,^20,39^ highlighting quinoidal resonance structure contributions to the ground state wavefunction.

## Conclusions

This work provides a detailed account of the transferability of electronic coupling with regard to exchange coupling and conductance. We have developed an electronic structure description of the functional relationship between *J*_D→A_ and *g*_mb_, which we observed to be nonlinear (*β*_g_/*β*_J_ ≠ 1). Plots of *J*_D→A_*vs. g*_mb_ for monomeric and dimeric bridges lie on different curves due to a scale invariance between these bridge types, which indicates a proportionate scaling of the exchange interaction, *J*_D→A_. Our *g*_mb_*vs. J*_D→A_ data yield *β*_g_/*β*_J_ = 1.15 for the phenylene series and *β*_g_/*β*_J_ = 1.58 for the thiophene series, and these ratios accurately replicate recently reported *β*-values for electron transport through oligothiophene and oligophenylene bridges.^59,60^ In addition, these *β*_g_/*β*_J_ ratios have been used to determine conductance tunneling barrier heights for oligo-phenylenes (Δ*E*_eff_ = 1555 cm^−1^) and oligo-thiophenes (Δ*E*_eff_ = 941 cm^−1^) that span electrodes in a transport geometry. This translates to a decrease in effective barrier heights Δ*E*_eff_ for magnetic exchange coupling of ∼25% for phenylenes and ∼60% for thiophenes when compared to conductance values using these same organic bridge fragments.

Tunneling gaps and bridge–bridge electronic coupling, ***H***_BB_, calculated from experimental data for the phenylene- and thiophene series provide a convincing explanation for the more pronounced *g*_mb_*vs. J*_D→A_ nonlinearity observed in the thiophene series compared to the phenylene series. Additionally, resonance Raman spectroscopy indicates that ***H***_BB_ may be enhanced by quinoidal resonance structure contributions in SQ–Bridge–NN biradicals relative to M–B–M devices for conductance. Namely, contributions from ECN resonance structures (Fig. 4) are expected to be more pronounced for **SQ–Thn–NN** than for **SQ–Phn–NN** or Au_*n*_–S–Bridge–S–Au_*n*_ constructs.

Resonance enhancement of the bridge quinoidal stretching mode illustrates the key bridge distortion that is the hallmark of a large ***H***_BB_, and the magnitude of this excited state distortion is proportional to the degree of SQ → B–NN charge transfer, which contributes to the magnitude of the magnetic exchange interaction in SQ–Bridge–NN biradicals. From a valence bond/resonance perspective, contributing ECN resonance structures accurately reflect both the spin- and charge distribution of low-lying SQ–Bridge–NN CT states, for which the corresponding quinoidal bridge-containing excited states in M–Bridge–M constructs lie far higher in energy. With respect to the importance of quinoidal resonance contributions, aromaticity has been shown to reduce conductance values in single molecule junctions.^65^ Critically, the quinoidal resonance structure contributions in Fig. 4 result in diminished aromaticity and contribute more to exchange than to conductance, leading to *γ* ≠ 1 and *β*_g_/*β*_J_ > 1 for these molecular bridges.

## Conflicts of interest

There are no conflicts to declare.

## Supplementary Material

SC-011-D0SC04350H-s001

SC-011-D0SC04350H-s002

## References

[cit1] Kirk M. L., Shultz D. A., Zhang J. Y., Dangi R., Ingersol L., Yang J., Finney N. S., Sommer R. D., Wojtas L. (2017). Chem. Sci..

[cit2] Tsuji Y., Movassagh R., Datta S., Hoffmann R. (2015). ACS Nano.

[cit3] Xia J. L., Capozzi B., Wei S. J., Strange M., Batra A., Moreno J. R., Amir R. J., Amir E., Solomon G. C., Venkataraman L., Campos L. M. (2014). Nano Lett..

[cit4] Venkatramani R., Wierzbinski E., Waldeck D. H., Beratan D. N. (2014). Faraday Discuss..

[cit5] Wierzbinski E., Venkatramani R., Davis K. L., Bezer S., Kong J., Xing Y. J., Borguet E., Achim C., Beratan D. N., Waldeck D. H. (2013). ACS Nano.

[cit6] Chen W. B., Widawsky J. R., Vazquez H., Schneebeli S. T., Hybertsen M. S., Breslow R., Venkataraman L. (2011). J. Am. Chem. Soc..

[cit7] Fung E. D., Gelbwaser D., Taylor J., Low J., Xia J. L., Davydenko I., Campos L. M., Marder S., Peskin U., Venkataraman L. (2019). Nano Lett..

[cit8] Ding W. D., Koepf M., Koenigsmann C., Batra A., Venkataraman L., Negre C. F. A., Brudvig G. W., Crabtree R. H., Schmuttenmaer C. A., Batista V. S. (2015). J. Chem. Theory Comput..

[cit9] Darancet P., Widawsky J. R., Choi H. J., Venkataraman L., Neaton J. B. (2012). Nano Lett..

[cit10] Aradhya S. V., Meisner J. S., Krikorian M., Ahn S., Parameswaran R., Steigerwald M. L., Nuckolls C., Venkataraman L. (2012). Nano Lett..

[cit11] Schneebeli S., Kamenetska M., Foss F., Vazquez H., Skouta R., Hybertsen M., Venkataraman L., Breslow R. (2010). Org. Lett..

[cit12] Kamenetska M., Quek S. Y., Whalley A. C., Steigerwald M. L., Choi H. J., Louie S. G., Nuckolls C., Hybertsen M. S., Neaton J. B., Venkataraman L. (2010). J. Am. Chem. Soc..

[cit13] Venkataraman L. (2008). Nat. Nanotechnol..

[cit14] Herrmann C. (2019). J. Phys. Chem. A.

[cit15] Venkataraman L., Klare J. E., Tam I. W., Nuckolls C., Hybertsen M. S., Steigerwald M. L. (2006). Nano Lett..

[cit16] Joachim C., Ratner M. A. (2005). Proc. Natl. Acad. Sci. U. S. A..

[cit17] Kirk M. L., Shultz D. A., Stasiw D. E., Lewis G. F., Wang G. B., Brannen C. L., Sommer R. D., Boyle P. D. (2013). J. Am. Chem. Soc..

[cit18] Kirk M. L., Shultz D. A., Stasiw D. E., Habel-Rodriguez D., Stein B., Boyle P. D. (2013). J. Am. Chem. Soc..

[cit19] Kirk M. L., Shultz D. A. (2013). Coord. Chem. Rev..

[cit20] Kirk M. L., Shultz D. A., Depperman E. C., Habel-Rodriguez D., Schmidt R. D. (2012). J. Am. Chem. Soc..

[cit21] Nitzan A. (2001). J. Phys. Chem. A.

[cit22] Ricks A. B., Solomon G. C., Colvin M. T., Scott A. M., Chen K., Ratner M. A., Wasielewski M. R. (2010). J. Am. Chem. Soc..

[cit23] Weiss E. A., Ahrens M. J., Sinks L. E., Gusev A. V., Ratner M. A., Wasielewski M. R. (2004). J. Am. Chem. Soc..

[cit24] Proppe J., Herrmann C. (2015). J. Comput. Chem..

[cit25] Herrmann C., Elmisz J. (2013). Chem. Commun..

[cit26] Stasiw D. E., Zhang J. Y., Wang G. B., Dangi R., Stein B. W., Shultz D. A., Kirk M. L., Wojtas L., Sommer R. D. (2015). J. Am. Chem. Soc..

[cit27] Nishizawa S., Hasegawa J., Matsuda K. (2014). Chem. Lett..

[cit28] Nishizawa S., Hasegawa J., Matsuda K. (2013). Chem. Phys. Lett..

[cit29] Nishizawa S., Hasegawa J., Matsuda K. (2013). J. Phys. Chem. C.

[cit30] Shinomiya M., Higashiguchi K., Matsuda K. (2013). J. Org. Chem..

[cit31] Tsuji Y., Hoffmann R., Strange M., Solomon G. C. (2016). Proc. Natl. Acad. Sci. U. S. A..

[cit32] Anderson P. W. (1950). Phys. Rev..

[cit33] Kramers H. A. (1934). Physica.

[cit34] Hay P. J., Thibeault J. C., Hoffmann R. (1975). J. Am. Chem. Soc..

[cit35] Kirk M. L., Shultz D. A., Depperman E. C., Brannen C. L. (2007). J. Am. Chem. Soc..

[cit36] Kirk M. L., Shultz D. A., Depperman E. C. (2005). Polyhedron.

[cit37] Steenbock T., Shultz D. A., Kirk M. L., Herrmann C. (2017). J. Phys. Chem. A.

[cit38] Kirk M. L., Shultz D. A. (2013). Coord. Chem. Rev..

[cit39] Kirk M. L., Shultz D. A., Habel-Rodriguez D., Schmidt R. D., Sullivan U. (2010). J. Phys. Chem. B.

[cit40] Shultz D. A., Vostrikova K. E., Bodnar S. H., Koo H. J., Whangbo M. H., Kirk M. L., Depperman E. C., Kampf J. W. (2003). J. Am. Chem. Soc..

[cit41] Shultz D. A., Kirk M. L., Zhang J. Y., Stasiw D. E., Wang G. B., Yang J., Habel-Rodriguez D., Stein B. W., Sommer R. D. (2020). J. Am. Chem. Soc..

[cit42] Kirk M. L., Shultz D. A. (2013). Coord. Chem. Rev..

[cit43] Frisch M. J., Trucks G. W., Schlegel H. B., Scuseria G. E., Robb M. A., Cheeseman J. R., Scalmani G., Barone V., Petersson G. A., Nakatsuji H., Li X., Caricato M., Marenich A., Bloino J., Janesko B. G., Gomperts R., Mennucci B., Hratchian H. P., Ortiz J. V., Izmaylov A. F., Sonnenberg J. L., Williams-Young D., Ding F., Lipparini F., Egidi F., Goings J., Peng B., Petrone A., Henderson T., Ranasinghe D., Zakrzewski V. G., Gao J., Rega N., Zheng G., Liang W., Hada M., Ehara M., Toyota K., Fukuda R., Hasegawa J., Ishida M., Nakajima T., Honda Y., Kitao O., Nakai H., Vreven T., Throssell K., Montgomery Jr J. A., Peralta J. E., Ogliaro F., Bearpark M., Heyd J. J., Brothers E., Kudin K. N., Staroverov V. N., Keith T., Kobayashi R., Normand J., Raghavachari K., Rendell A., Burant J. C., Iyengar S. S., Tomasi J., Cossi M., Millam J. M., Klene M., Adamo C., Cammi R., Ochterski J. W., Martin R. L., Morokuma K., Farkas O., Foresman J. B. and Fox D. J., Gaussian 09, Revision A.02, Gaussian, Inc., Wallingford CT, 2016

[cit44] Smidstrup S., Markussen T., Vancraeyveld P., Wellendorff J., Schneider J., Gunst T., Verstichel B., Stradi D., Khomyakov P. A., Vej-Hansen U. G., Lee M.-E., Chill S. T., Rasmussen F., Penazzi G., Corsetti F., Ojanpera A., Jensen K., Palsgaard M. L. N., Martinez U., Blom A., Brandbyge M., Stokbro K. (2019). J. Phys.: Condens. Matter.

[cit45] Brandbyge M., Mozos J.-L., Ordejon P., Taylor J., Stokbro K. (2002). Phys. Rev. B: Condens. Matter Mater. Phys..

[cit46] Soler J. M., Artacho E., Gale J. D., Garcia A., Junquera J., Ordejon P., Sanchez-Portal D. (2002). J. Phys.: Condens. Matter.

[cit47] Büttiker M., Imry Y., Landauer R., Pinhas S. (1985). Phys. Rev. B: Condens. Matter Mater. Phys..

[cit48] Van Dyck C., Ratner M. A. (2015). Nano Lett..

[cit49] Taylor J., Guo H., Wang J. (2001). Phys. Rev. B: Condens. Matter Mater. Phys..

[cit50] Depperman E. C., Bodnar S. H., Vostrikova K. E., Shultz D. A., Kirk M. L. (2001). J. Am. Chem. Soc..

[cit51] Van Dyck C., Ratner M. A. (2017). J. Phys. Chem. C.

[cit52] Van Dyck C., Geskin V., Cornil J. (2014). Adv. Funct. Mater..

[cit53] Priyadarshy S., Skourtis S. S., Risser S. M., Beratan D. N. (1996). J. Chem. Phys..

[cit54] Yoshizawa K., Tada T., Staykov A. (2008). J. Am. Chem. Soc..

[cit55] Tsuji Y., Staykov A., Yoshizawa K. (2011). J. Am. Chem. Soc..

[cit56] Gray H. B., Winkler J. R. (2005). Proc. Natl. Acad. Sci. U. S. A..

[cit57] Wenger O. S. (2011). Acc. Chem. Res..

[cit58] Wenger O. S. (2011). Chem. Soc. Rev..

[cit59] Wold D. J., Haag R., Rampi M. A., Frisbie C. D. (2002). J. Phys. Chem. B.

[cit60] Capozzi B., Dell E. J., Berkelbach T. C., Reichman D. R., Venkataraman L., Campos L. M. (2014). J. Am. Chem. Soc..

[cit61] Pauling L., Wheland G. W. (1933). J. Chem. Phys..

[cit62] Wheland G. W., Pauling L. (1935). J. Am. Chem. Soc..

[cit63] Pauling L., The Nature of the Chemical Bond, Cornell University Press, Ithaca, NY, 3rd edn, 1960

[cit64] Weinhold F. and Landis C. R., Valency and bonding: a natural bond orbital donor–acceptor perspective, Cambridge University Press, Cambridge, UK; New York, 2005

[cit65] Chen W. B., Li H. X., Widawsky J. R., Appayee C., Venkataraman L., Breslow R. (2014). J. Am. Chem. Soc..

