# Supplementary material for: Transferrable property relationships between magnetic exchange coupling and molecular conductance
Source: Chem Sci. 2020 Oct 8;11(42):11425–34. doi: 10.1039/d0sc04350h (PMC8162509; doi:10.1039/d0sc04350h)
Supplement: SC-011-D0SC04350H-s001 [file SC-011-D0SC04350H-s001.pdf]

Electronic Supplementary Material (ESI) for Chemical Science.
This journal is © The Royal Society of Chemistry 2020

Supporting Information

Transferrable Property Relationships Between Magnetic Exchange Coupling and Molecular Conductance

Martin L. Kirk* Ranjana Dangi, Diana Habel-Rodriquez, Jing Yang, David A. Shultz,* and Jinyuan Zhang

General Considerations Reagents and solvents were purchased from commercial sources and used as received unless otherwise noted. ^1H and ^{13}C NMR spectra were recorded on a Varian Mercury 400 MHz or a Varian Mercury 300 MHz spectrometer at room temperature. ^1H and ^{13}C chemical shifts are listed in parts per million (ppm) and are referenced to residual protons or carbons of the deuterated solvents, respectively. EPR spectra were recorded on an IBM ER200D-SRC EPR spectrometer in CH_2Cl_2 solution. Infrared spectra were recorded on a Brüker Vertex 80v spectrometer with Brüker Platinum ATR attachment. Mass spectra were obtained at the NCSU Mass Spectrometry Facility located in the Department of Chemistry. Compound **1**,[1] compound **2**,[2] 2,3-dimethyl-2,3-bis(hydroxyamino)butane (**BHA**),[3,4] and **Tp^{Cum,Me}Zn(OH)**[5] were prepared as previously reported.

Scheme S1

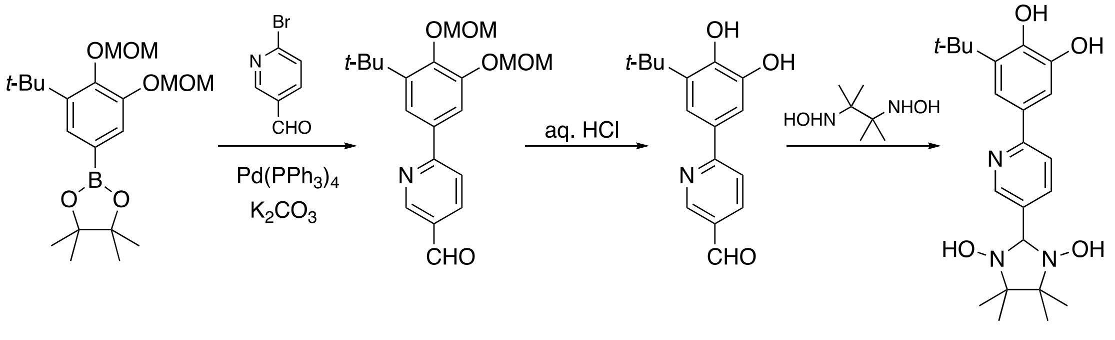

1 **2** **3 (82%)** **4** (64%) **5**

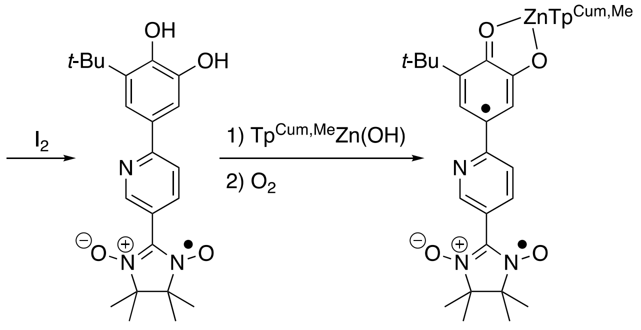

6 (44%) **SQ-NPy-NN** (38%)

6-(3-(*tert*-Butyl)-4,5-bis(methoxymethoxy)phenyl)picolinaldehyde (3). To a 50 mL Schlenk flask were added compound **2** (0.20 g, 1.08 mmol) and compound **1** (0.37 g, 0.98 mmol), which was then transferred to the glove box where $Pd(PPh_3)_4$ (0.08 g, 0.1 mmol) was added with 25 mL THF. The Schlenk flask was transferred out of the

glovebox and 1.08 mL (2.16 mmol) of degassed 2 M K_2CO_3 solution were added. The reaction flask was fitted with a condenser under nitrogen and heated to reflux for 60 h. The reaction was then cooled, filtered into a separatory funnel and washed with 20 mL of saturated NaCl solution twice. The organic layers were collected and the solvent removed under reduced pressure. The crude product was purified by column chromatography (SiO_2, 25% ethyl acetate in hexanes) to yield compound **3** (0.29 g, 82%). ^{1}H NMR (300 MHz, $CDCl_3$, δ): 10.11 (s, 1H), 9.08 (d, J = 2.1 Hz, 1H), 8.19 (dd, J = 2.1 Hz, J = 8.1 Hz, 2H), 7.79 (d, J = 2.4 Hz, 1H), 7.76 (d, J = 2.4 Hz, 1H), 5.29 (s, 2H), 5.28 (s, 2H), 3.68 (s, 2H), 3.58 (s, 2H), 1.50 (s, 9H). ^{13}C NMR (100 MHz, $CDCl_3$, δ): 193.19, 151.73, 150.91, 148.73, 147.36, 144.48, 140.89, 135.06, 131.45, 121.84, 119.99, 113.89, 99.36, 95.66, 57.81, 56.55, 35.54, 30.55. IR (solid) ν_{max} (cm^{-1}): 1710 (s, C=O). The chromatographed product was used for next step without further purification.

6-(3-(*tert*-Butyl)-4,5-dihydroxyphenyl)picolinaldehyde (4). To a 100 mL round bottom flask were added **3** (0.72 g, 2.00 mmol) and 50 mL ethyl acetate. The mixture was added 10 mL 4 M HCl solution in EtOAc and stirred for 30 min. To the reaction was then added saturated $NaHCO_3$ solution until pH of the aqueous phase was 9. The mixture was then transferred to a separatory funnel. The organic layer was washed once with saturated aq. $NaHCO_3$ solution and followed by another wash of saturated aq. NaCl solution. The organic layer was evaporated under reduced pressure. The crude product was purified by column chromatography (SiO_2, 50% ethyl acetate in hexanes) to yield compound **4** (0.32 g, 60%). ^{1}H NMR (300 MHz, $CDCl_3$, δ): 10.07 (s, 1H), 8.98 (d, J = 1.8 Hz, 1H), 8.14 (dd, J = 8.4 Hz, J = 1.8 Hz, 1H), 7.71 (d, J = 8.4 Hz, 1H), 7.60 (d, J = 2.1 Hz, 1H), 7.40 (d, J = 2.1 Hz, 1H), 1.46 (s, 9H). ^{13}C NMR (100 MHz, DMSO-d_6, δ): 192.43, 161.91, 152.59, 147.64, 146.15, 137.26, 136.53, 129.53, 127.81, 119.79, 117.57, 112.53, 35.22, 30.02. IR (solid) ν_{max} (cm^{-1}): 3244 (br, OH), 1689 (s, C=O). The chromatographed product was used for next step without further purification.

2-(6-(3-(tert-butyl)-4,5-dihydroxyphenyl)pyridin-2-yl)-4,4,5,5-tetramethylimidazolidine-1,3-diol (5). To a 25 mL Schlenk flask were added compound **4** (0.32 g, 1.19 mmol) and 2,3-dimethyl-2,3-bis(hydroxyamino)butane (**BHA**, 0.26 g, 1.79 mmol). The flask was pump purged three times and filled with N_2. Then 3 mL anhydrous MeOH was added to the reaction flask via syringe. The reaction flask was stirred in the dark at room temperature for 90 h, and then solvent was removed under reduced pressure. The crude product **5** was collected and used for next step without further purification.

2-(6-(3-(*tert*-Butyl)-4,5-dihydroxyphenyl)pyridin-2-yl)-4,4,5,5-tetramethyl-4,5-dihydroimidazol-3-oxide-1-oxyl (6). To a 250 mL round bottom flask, compound **5** (0.48 g, 1.19 mmol) was added with 60 mL CH_2Cl_2, 40 mL buffer (pH = 7), and then cooled to 0 °C. To a 60 mL separatory funnel, 0.45 g (1.79 mmol) I_2 was added to 20 mL CH_2Cl_2 and the solution was added dropwise to the stirring reaction mixture at 0

°C. After all of the I_2 was added, the reaction was stirred at 0 °C for 1 h and then warmed to room temperature. The reaction mixture was transferred to a separatory funnel containing 100 mL aq. phosphate buffer (pH = 7). The organic layer was washed once with 5 mL saturated $Na_2S_2O_3$ solution followed by another wash of saturated aq. NaCl solution. The organic layer was dried over Na_2SO_4 and the solvent removed under reduced pressure to yield 0.37 g (79%) of compound **6** as a brown solid. IR (solid) ν_{max} (cm^{-1}): 3083 (br, OH). EPR (X-Band, 298 K): a_N = 7.40 G (1:2:3:2:1). The crude product **6** was collected and used for next step without further purification.

$Tp^{Cum,Me}$Zn(SQ-NPy -NN) (SQ-NPy-NN). To an oven dried 25 mL Schlenk flask, compound **6** (0.10 g, 0.25 mmol) was added with **$Tp^{Cum,Me}$Zn(OH)** (0.18 g, 0.26 mmol) and pump purged with nitrogen three times. Then 10 mL anhydrous CH_2Cl_2 was added to the Schlenk flask. In the dark, the reaction was stir for 1 h under nitrogen then opened to air and stirred for additional 12 h. The solvent was removed under reduced pressure and the product purified by column chromatography (basic alumina, 30% ethyl acetate in hexanes) to yield **SQ-NPy-NN** (0.15 g, 56%). Crystals were grown from slow diffusion of *n*-hexane vapor into concentrated toluene solution of **SQ-NPy-NN**. IR (solid) ν_{max} (cm^{-1}): 2541 (w, BH). EPR (~0.2 mM in CH_2Cl_2): apparent a_N = 3.70 G. Mass spectrometry (*m*/*z*): calculated for $C_{61}H_{76}N_9O_4BZn$ $(M+H)^+$: 1173.540, found: 1173.54021 $(M+H)^+$

X-ray Crystallography. A lustrous dark purple block-like specimen of $C_{73}H_{100}BN_9O_4Zn$, approximate dimensions 0.240 mm x 0.260 mm x 0.280 mm, was used for the X-ray crystallographic analysis. The X-ray intensity data were measured on a Bruker-Nonius X8 Kappa APEX II system equipped with a fine-focus sealed tube (MoKα, λ = 0.71073 Å) and a graphite monochromator.

The total exposure time was 14.59 hours. The frames were integrated with the Bruker SAINT software package using a narrow-frame algorithm. The integration of the data using a monoclinic unit cell yielded a total of 12541 reflections to a maximum θ angle of 23.34° (0.90 Å resolution), of which 12541 were independent (average redundancy 1.000, completeness = 99.5%, R_{int} = 7.87%, R_{sig} = 9.30%) and 9295(74.12%) were greater than $2\sigma(F^2)$. The final cell constants of a = 17.5685(15) Å, b = 17.8590(15) Å, c = 22.7631(18) Å, β = 105.077(3)°, volume = 6896.2(10) $Å^3$, are based upon the refinement of the XYZ-centroids of 9264 reflections above 20 σ(I) with 4.480° < 2θ < 44.68°. Data were corrected for absorption effects using the multi-scan method (SADABS). The ratio of minimum to maximum apparent transmission was 0.866. The calculated minimum and maximum transmission coefficients (based on crystal size) are 0.8940 and 0.9080.

The structure was solved and refined using the Bruker SHELXTL Software Package, using the space group P 1 21/n 1, with Z = 4 for the formula unit, $C_{73}H_{100}BN_9O_4Zn$. The final anisotropic full-matrix least-squares refinement on F^2 with 814 variables converged at R1 = 7.01%, for the observed data and wR2 = 17.97% for all data. The goodness-of-fit was 1.053. The largest peak in the final

difference electron density synthesis was 0.508 $e^-/Å^3$ and the largest hole was -0.495 $e^-/Å^3$ with an RMS deviation of 0.088 $e^-/Å^3$. On the basis of the final model, the calculated density was 1.198 g/cm^3 and F(000), 2672 e^-.

Table S1. Sample and crystal data for rds175 (SQ-NPy-NN).

Identification code	rds211	
Chemical formula	$C_{73}H_{100}BN_9O_4Zn$	
Formula weight	1243.79	
Temperature	110(2) K	
Wavelength	0.71073 Å	
Crystal size	0.240 x 0.260 x 0.280 mm	
Crystal habit	Lustrous dark purple block	
Crystal system	monoclinic	
Space group	P 1 21/n 1	
Unit cell dimensions	a = 17.5685 (15) Å	α = 90°
	b = 17.8590(15) Å	β = 105.077(3)°
	c = 22.7631(18) Å	γ = 90°
Volume	6896.2(10) Å^3	
Z	4	
Density (calculated)	1.198 g/cm^3	
Absorption coefficient	0.410 mm^{-1}	
F(000)	2672	

Table S2. Data collection and structure refinement for rds211 (SQ-PyN-NN).

Diffractometer	Bruker-Nonius X8 Kappa APEX II
Radiation source	fine-focus sealed tube (MoKα, λ = 0.71073 Å)
Theta range for data collection	1.66 to 23.34°
Reflections collected	12541
Independent reflections	12541 [R(int) = 0.0787]
Coverage of independent reflections	99.5%
Absorption correction	multi-scan
Max. and min. transmission	0.9080 and 0.8940
Structure solution technique	direct methods
Structure solution program	SHELXS-97 (Sheldrick, 2008)
Refinement method	Full-matrix least-squares on F^2
Refinement program	SHELXL-2018/3 (Sheldrick, 2018)
Function minimized	$\Sigma w(F_o^2 - F_c^2)^2$
Data / restraints / parameters	12541 / 66 / 814
Goodness-of-fit on F^2	1.053

Δ/σ_{max}	0.001
Final R indices	9295 data; I>:R1 = 0.0701, wR2 = 0.1581
	all data R1 = 0.1103, wR2 = 0.1797
Weighting scheme	$w=1/[\sigma^2(F_o^2)+(0.0732P)^2+14.2396P]$ where $P=(F_o^2+2F_c^2)/3$
Largest diff. peak and hole	0.508 and -0.495 eÅ^{-3}
R.M.S. deviation from mean	0.088 eÅ^{-3}

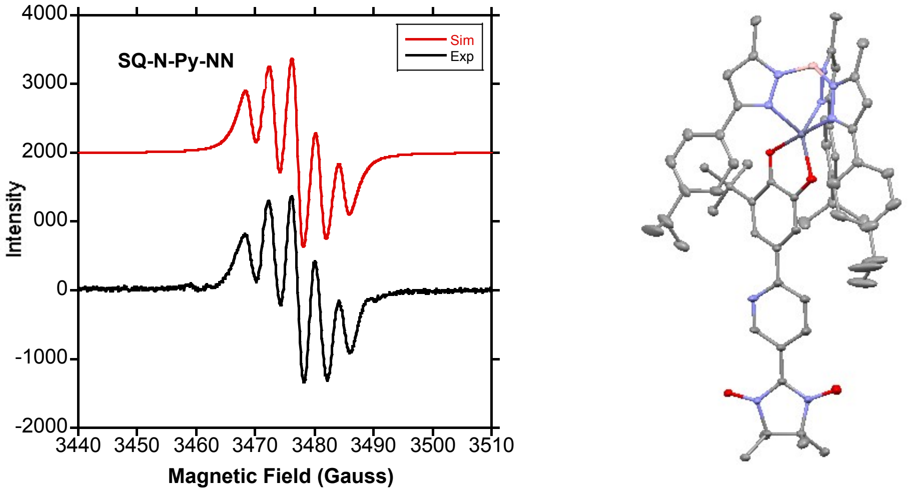

Figure S1. Experimental and simulated EPR spectra of **SQ-NPy-NN**, a_N = 3.88 (left). Crystal structure of **SQ-NPy-NN** (right).

Magnetometry. Magnetic susceptibility measurements were collected on a Quantum Design MPMS-XL7 SQUID magnetometer with an applied field of 0.7 T. A crystalline sample (~20 mg) was loaded into a gelcap/straw sample holder and mounted to the sample rod with Kapton tape for variable temperature measurements. Raw data were corrected with Pascal's constants as a first approximation for molecular diamagnetism followed by a straight-line correction to all data for diamagnetic response of sample container where the slope of the line represents the residual diamagnetic correction. Fits were achieved using a Curie Weiss expression as described elsewhere.[6] The paramagnetic susceptibility-temperature product (χ_{para}•T) vs. temperature plot is shown in Figure S2 along with fit parameters.

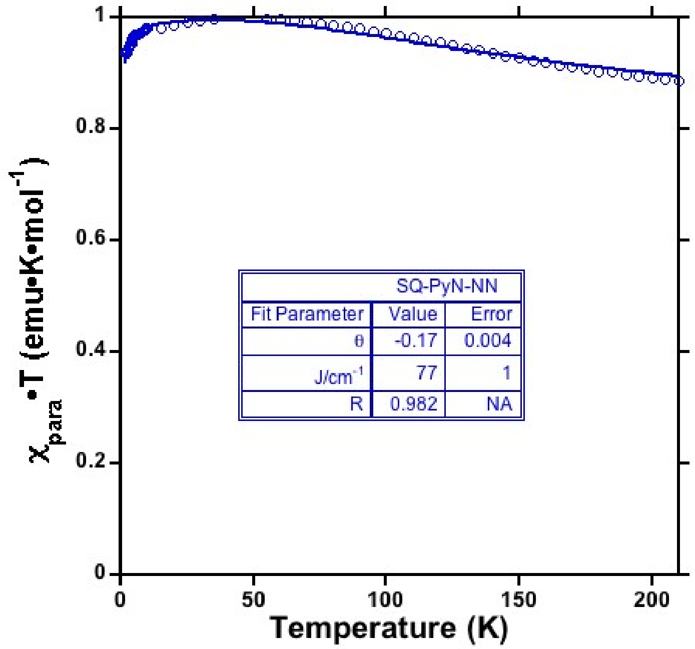

Figure S2. Magnetic susceptibility $\chi_{para}T$ vs. T plot for crystalline sample of **SQ-NPy-NN** with fit parameters.

Transmission Computations.

Computed phenylene I-V plot and associated MPSH HOMO.

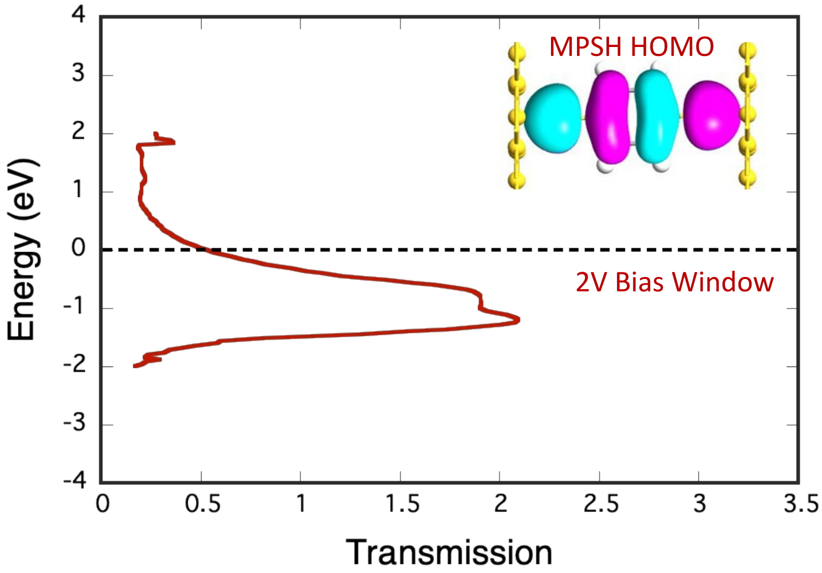

Figure S3. Computed zero-bias energy vs. transmission plot for S-**Ph**-S connected to Au electrodes. A 2V bias window is depicted in yellow and the Fermi energy is shown as a dashed line. ***Inset:*** Molecular projected self-consistent Hamiltonian (MPSH) state that dominantly contributes to g_{mb}, which is primarily comprised of the **Ph** HOMO.

Bulk configuration convergence criteria.

Bulk configurations were optimized by using the advanced relaxation method. Relaxation of the electrodes was performed first. After constructing the bulk configuration, the electrodes were relaxed, and the geometry optimized molecules were attached to the gold electrodes. Then, the bulk configuration was optimized by setting the force tolerance to 0.01 eV/Å, the stress tolerance to 0.001 eV/Å^3, the damping factor to 0.05, the density mess cut-off to 75 Ha, and we unchecked the constrain lattice parameters in the "z" direction with a maximum number of optimization steps set to 500. During optimization, both the electrodes and the molecules were set rigid by using the add constraint feature, allowing the relaxation of the molecule-electrode junction only during geometry optimization of the bulk configuration. (see reference: quantumwise tutorial for advance device relaxation).

Cartesian Coordinates for the bridge molecules used in conductance calculations

Phenylene

```
XYZ.Cordinates
C       1.397939562    -0.001119065    -0.000010115
C       0.693118806    -1.195799970    -0.000045220
C      -0.693114932    -1.195801589    -0.000063028
C      -1.397938826    -0.001125128    -0.000003765
C      -0.691158368     1.196090769     0.000062068
C       0.691154373     1.196092144     0.000038340
H       1.214354977    -2.139050796    -0.000063957
H      -1.214348722    -2.139054490    -0.000153272
H      -1.218828462     2.136055827     0.000177157
H       1.218821688     2.136059018     0.000090436
S      -3.179138386     0.077297240    -0.000134772
S       3.179138741     0.077297452     0.000018232
H      -3.417357722    -1.228772422     0.002100056
H       3.417348896    -1.228775179    -0.000155464
```

Thiophene

```
XYZ.Coordinates
C      -1.244848970    -0.202600118     0.000013724
C      -0.720278442    -1.440591973     0.000059994
C       0.720278452    -1.440591920    -0.000103905
C       1.244849007    -0.202600092    -0.000027843
S      -0.000000020     1.014468596     0.000040756
H      -1.309766942    -2.340898308     0.000093115
H       1.309766964    -2.340898277    -0.000193765
S       2.939323909     0.312095656    -0.000102952
S      -2.939323888     0.312095724     0.000027284
H       3.461313862    -0.909228809     0.001589931
H      -3.461314169    -0.909229624    -0.000582492
```

Pyridine

XYZ.Coordinates

C	-1.353502669	0.006825866	-0.000127670
C	-0.691975449	1.230346867	-0.000116278
C	0.688722412	1.226132029	0.000020887
C	1.367866921	0.015158508	0.000137132
C	0.601108365	-1.141250003	0.000094511
N	-0.717237096	-1.143990728	-0.000030443
H	1.224428872	2.161568330	0.000032864
H	-1.234484525	2.159382871	-0.000206417
H	1.075498671	-2.110217748	0.000193382
S	-3.125927122	-0.147919233	-0.000317340
S	3.142841722	-0.135621361	0.000316849
H	-3.414590750	1.149124594	-0.000415147
H	3.425856325	1.161446942	0.000564785

Bicycloctane

XYZ.Cordinates

C	-1.302566602	0.008087992	-0.000184200
C	-0.774627263	0.783733737	1.217725762
C	1.302574318	0.008102652	0.000123522
C	-0.772317875	0.668524801	-1.283406671
H	-1.221028638	1.651390227	-1.406561605
H	-1.226631212	0.393695460	2.124736377
C	-0.772589946	-1.435106278	0.070050599
H	-1.075871720	1.826793673	1.138048608
H	-1.078169408	0.075362981	-2.141080392
H	-1.222541058	-2.034681596	-0.717230936
H	-1.070258044	-1.879607579	1.016667798
C	0.774627342	0.784319488	-1.217442330
C	0.772612039	-1.435056784	-0.070749034
C	0.772308731	0.667911130	1.283680901
H	1.226636160	0.394739175	-2.124655603
H	1.075855956	1.827343298	-1.137287730
H	1.222663991	-2.034973146	0.716224055
H	1.070302997	-1.879085741	-1.017590816
H	1.220977488	1.650738641	1.407300665
H	1.078178753	0.074380855	2.141103734
S	3.136622545	-0.087730043	0.001684292
S	-3.136631991	-0.087735446	-0.001631028

H	3.388046049	1.219133400	-0.008687298
H	-3.388134665	1.219117726	0.009369599

Pyridine-Thiophene

XYZ.Coordinates

C	-0.70236200	0.08656500	0.03398600
C	-1.50906400	1.19820500	-0.28329800
C	-3.45223600	0.02515900	-0.02369000
C	-1.37043600	-1.11505800	0.31883700
H	-0.80471000	-2.00630600	0.58117600
H	-1.03650000	2.14416500	-0.54721400
C	0.75869600	0.20805600	0.05792000
C	1.52180600	1.32406600	0.32245000
S	1.79859700	-1.16207400	-0.28856600
C	2.92524800	1.08636300	0.25434900
H	1.08743900	2.28250800	0.58780000
C	3.22920200	-0.21014300	-0.06182700
H	3.67634100	1.84708200	0.44139500
C	-2.76119900	-1.14811500	0.28336800
H	-3.30304200	-2.06457100	0.50185100
N	-2.84249000	1.18435200	-0.30475100
S	-5.23170129	0.04243386	-0.06375081
S	4.83183466	-0.96035996	-0.25455233
H	-5.63796574	0.53557909	-1.20736655
H	5.76244136	-0.06736238	-0.02514769

Bithiophene

XYZ.Coordinates

C	-0.708375176	-0.192817967	-0.157582825
C	-1.249805625	-1.401705277	-0.436746846
C	-2.679663978	-1.410975647	-0.350993281
C	-3.195003591	-0.211299128	-0.012858524
S	-1.941514348	0.962073772	0.246699589
H	-0.666970337	-2.260690008	-0.720077539
H	-3.287453989	-2.276862628	-0.544511255
C	0.707831732	0.187373665	-0.165680998
C	1.245638269	1.400140256	-0.434375068
S	1.946151602	-0.973422814	0.205532893
C	2.675244512	1.414131560	-0.344091736
H	0.659597051	2.261575745	-0.703128569
C	3.193980480	0.215743322	-0.006892942

H	3.280291469	2.283772402	-0.529207809
S	-4.900990274	0.168408468	0.260562255
S	4.909627261	-0.194895128	0.123044699
H	-5.133387205	0.828440091	-0.872489514
H	4.996455379	-0.234409152	1.451317020

Biphenylene

XYZ.Coordinates

C	-2.848219521	1.111062436	0.446082048
C	-1.465169488	1.107430830	0.442722740
C	-0.745157853	-0.000344463	-0.000453491
C	-1.465181464	-1.106502309	-0.442433740
C	-2.850230426	-1.111709625	-0.444875321
C	-3.553632627	-0.000991742	0.001043374
H	-3.377811449	1.978651817	0.805035883
H	-0.939277996	1.972678591	0.811699299
H	-0.940216894	-1.972072842	-0.812236662
H	-3.373977422	-1.982201485	-0.804444893
C	0.744945139	0.000206749	-0.000106095
C	1.464296351	1.104424013	-0.451921495
C	1.465080724	-1.103467377	0.450104093
C	2.848090042	1.105602528	-0.460868136
H	0.938009468	1.965584520	-0.829705036
C	2.849664879	-1.106202160	0.456831176
H	0.940213238	-1.965932121	0.827215728
C	3.553154055	-0.001211605	-0.003925331
H	3.378078848	1.964957328	-0.837864738
H	3.374844072	-1.969093383	0.832256972
S	-5.334170224	0.073618418	0.024096014
S	5.334958592	0.077412518	-0.000859766
H	-5.573605255	-1.171363295	-0.369860071
H	5.575290949	-1.227487738	0.052924620

References

1. Shultz, D. A.; Hollomon, M. G., Preparation and EPR Spectroscopic Investigation of Conjugated Oligomers Containing Semiquinone Repeat Units. *Chem. Mater.* **2000,** *12*, 580-585.
2. Rocha, S. V.; Finney, N. S., Synthesis and Evaluation of 2,5-Linked Alternating Pyridine-Thiophene Oligomers. *Org. Lett.* **2010,** *12* (11), 2598-2601.
3. Osiecki, J. H.; Ullman, E. F., Studies of free radicals. I. .alpha.-Nitronyl nitroxides, a new class of stable radicals. *J. Am. Chem. Soc.* **1968,** *90*, 1078-1079.
4. Shimono, S.; Tamura, R.; Ikuma, N.; Takimoto, T.; Kawame, N.; Tamada, O.; Sakai, N.; Matsuura, H.; Yamauchi, J., Preparation and Characterization of New Chiral Nitronyl Nitroxides Bearing a Stereogenic Center in the Imidazolyl Framework. *J. Org. Chem.* **2004,** *69*, 475–481.
5. Ruf, M.; Vahrenkamp, H., Small Molecule Chemistry of the Pyrazolylborate—Zinc Unit $Tp^{Cum,Me}Zn$. *Inorg. Chem.* **1996,** *35*, 6571-6578.
6. Kirk, M. L.; Shultz, D. A.; Stasiw, D. E.; Habel-Rodriguez, D.; Stein, B.; Boyle, P. D., Electronic and Exchange Coupling in a Cross-Conjugated D-B-A Biradical: Mechanistic Implications for Quantum Interference Effects. *J. Am. Chem. Soc.* **2013,** *135*, 14713-14725.
